# Optimal global spending for group A Streptococcus vaccine research and development

**DOI:** 10.1038/s41541-023-00646-6

**Published:** 2023-04-25

**Authors:** Daniel Tortorice, Maddalena Ferranna, David E. Bloom

**Affiliations:** 1 Department of Economics and Accounting, College of the Holy Cross, Worcester, MA USA; 2 Department of Pharmaceutical and Health Economics, University of Southern California School of Pharmacy, Los Angeles, CA USA; 3 Department of Global Health and Population, Harvard T.H. Chan School of Public Health, Boston, MA USA

**Keywords:** Bacterial infection, Epidemiology

## Abstract

Group A Streptococcus (Strep A) leads to 600,000 deaths and 600 million cases of pharyngitis annually. Although long a promising target for vaccine development, how much funding should be allocated to develop a Strep A vaccine is unclear. We aim to calculate the optimal amount of global spending for Strep A vaccine development, the resulting benefits, and the social rate of return on this spending. We develop a model of optimal spending, from a global societal perspective, on research and development (R&D) for vaccines and treatments. The model takes as inputs total harm from the disease, the probability an R&D project succeeds, the cost of a project, and the fraction of total harm a success alleviates. Based on these inputs the model outputs an optimal amount of spending and a rate of return. We calibrate the model for Strep A. Optimal spending is estimated to be 2020 USD33 billion. This spending leads to 2020 USD1.63 trillion in benefits and a real return of 22.3% per year for thirty years. Sensitivity shows an optimal spending range of 15.9 billion to 58.5 billion, a benefits range of 1.6 trillion to 37.9 trillion, and a return range of 18.0–48.2%. Investment in a Strep A vaccine could create enormous benefits for comparatively little cost. It represents one of the highest return uses of public spending. Policy can promote Strep A vaccine development through direct funding of projects and by promoting financial mechanisms that allow the private sector to diversify its R&D investment.

## Introduction

Group A Streptococcus (Strep A) is one of the deadliest pathogens in the world, leading to more than 600,000 deaths per year. Moreover, even in countries where antibiotic treatment is readily available, Strep A has a considerable disease burden contributing more than 600 million cases of pharyngitis per year along with substantial morbidity from cellulitis, invasive disease, and skin infections^1^.

Since the U.S. Food and Drug Administration lifted the ban on human subject testing of Strep A vaccines in 2004, Strep A has become a promising target for vaccine development^2^. However, the question of how much research and development (R&D) funding should be allocated to produce such a vaccine remains unanswered.

To address this question, we develop a mathematical model that allows us to calculate optimal R&D spending for treatments and vaccines against various pathogens. We then calibrate the model for Strep A using parameters from the medical and economics literatures. Using the model, we calculate optimal global spending on R&D for Strep A vaccines. We take the perspective of a supranational organization that can allocate funding for projects seeking to develop a Strep A vaccine. As such the organization accounts for total global harm caused by Strep A.

Because not all projects will succeed and not all successes will address all harm from Strep A, funding multiple projects will be optimal. Therefore, we first ask how many projects the organization should fund and then calculate the amount needed to fund all these projects. Next, we calculate the benefit of this funding, based on the amount of future harm from Strep A disease that the successful development of Strep A vaccines will prevent globally. Finally, we calculate a social rate of return on this investment, a measure of the monetary value of this expected harm reduction divided by the cost of this funding.

## Results

### Model mechanism

**Fig. 1:**
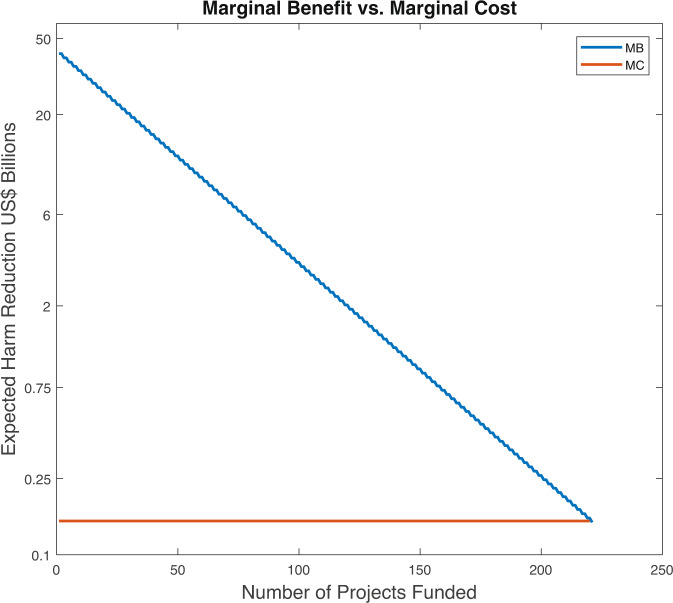
Calibrated model. The vertical axis uses a log scale. Monetary values in 2020 USD.

Figure 1 illustrates a version of the model calibrated as described in the methods section. The orange line (MC) represents the cost of funding a project. This cost is constant at USD150 million and does not depend on the number of projects funded. In contrast, the blue line (MB) represents the benefit of funding the next highest value project given the number of projects that have been funded in the past. This line slopes downward because the more projects are funded the more likely a successful vaccine (or more) will be developed. As a result, less harm from Strep A remains, and therefore, an additional project is less beneficial. For example, the first project has an expected benefit of about USD50 billion. This benefit is a product of the estimated harm caused by Strep A, the expected harm alleviated by a successful vaccine project, and the likelihood this first project will succeed. The second project has a smaller expected benefit because with some probability the first project will succeed and less harm will remain from Strep A that a second success can reduce.

As long as the MB line is above the MC line, the organization should continue to fund projects as the expected benefit of doing so is larger than the cost. The organization will continue funding projects until it reaches the 221st project. At this point, enough harm from Strep A is expected to be reduced that funding additional projects is no longer worthwhile.

Figure 1 has several important implications. First, if the measured total global harm from Strep A increases, the MB line will shift out. Consequently, optimal spending to develop a Strep A vaccine will be higher. Second, other parameters, like the probability of success or the fraction of harm reduced from a success, shift and change the slope of the MB line. Therefore, these parameters have an ambiguous effect on the number of projects to fund. Finally, if we measure social surplus as total expected benefits minus total costs then the area of the triangle formed by the y-axis, the MC line, and the MB line equals the social surplus.

### Optimal R&D results

Table 1 provides our numerical results for the baseline calibration and various sensitivity analyses. Under the parameters described in the methods section funding 220 projects at a cost of USD33 billion (in 2020 USD) is optimal. Strikingly, the social surplus generated by this investment is USD1.63 trillion. These benefits are large, equivalent to about 2% of annual world economic output (gross domestic product). Calculating a rate of return on this investment, assuming that the benefits accrue over a 30-year period beginning 10 years after the initial investment, leads to an annual return on investment of 22.3% per year for 30 years.

**Table 1:** Optimal spending.

Baseline calibration	Projects funded	Optimal spending (USD 2020)	Social surplus (USD 2020)	Internal rate of return
	220	33.0 billion	1.63 trillion	22.3%
*Sensitivity*				
Harm reduction = 70%	106	15.9 billion	1.65 trillion	28.5%
Success probability = 5%	272	40.8 billion	1.62 trillion	20.7%
Total Strep A harm 2x	248	37.2 billion	3.29 trillion	27.1%
Harm = 42.2 trillion	342	51.3 billion	37.9 trillion	48.2%
Require four approaches	388	58.2 billion	1.60 trillion	18.0%
Harm reduction = 15%	390	58.5 billion	1.60 trillion	18.0%
Constant Global VSL	272	40.8 billion	6.08 trillion	31.7%

This table contains the main results of the model. Internal rate of return is calculated assuming a 10-year delay before harm reduction begins and assuming harm reduction spreads out evenly over 30 years. All monetary values are in 2020 USD.

If we have underestimated the harm that a successful vaccine can reduce and consider the possibility that a successful vaccine will reduce 70% of the harm instead of 30%, then we would require less spending on vaccine R&D, USD15.9 billion; however, social surplus would remain almost unchanged. Therefore, the rate of return on this investment increases to 28.5%.

If projects are less likely to result in a successful vaccine, a 5% probability versus a 15% probability, then more projects need to be funded. We obtain this result because more projects are required to reduce expected harm to the point where future projects are no longer beneficial. The rate of return on investment, in this case, is 20.7%.

If we have underestimated total harm from Strep A, we will underestimate optimal spending. We consider a doubling of the harm caused by Strep A. In this case the number of projects funded and spending rise, though by a smaller factor than the increase in estimated harm. However, the benefits of the spending double and the returns to investment rise to 27.1%. Other estimates of Strep A harm are even larger. For example, using a static cohort epidemiological model^3^, the global burden of Strep A for the 2022–2051 birth cohorts is estimated at USD42.2 trillion assuming a 3% discount rate and a value per DALY equal to three times global per capita gross domestic product (personal communication with Maddalena Ferranna). Using this harm estimate we find optimal spending to be USD51.3 billion with a rate of return of 48.2%.

Next, we examine the case where we require four approaches to address all the harm associated with Strep A. In this case we should fund almost 400 projects at a cost of about USD60 billion. Social surplus differs little from the baseline case. Therefore, the return to investment falls to a still quite substantial 18.0% per year for 30 years.

Because harm reduction also depends on vaccine coverage, we consider a scenario where the vaccine reduces substantially less harm. In this case, the vaccine reduces only 15% of Strep A harm vs. 30%. We find in this case that we should fund 390 projects and the rate of return on R&D investment falls to 18%.

In measuring disease harm in our baseline case, we use a VSLY approach to assign a monetary value of disease harm. VSLYs vary by income and therefore across countries. We find this assumption relevant from the point of view of a national policy maker deciding how much to spend on treating a disease within the country but acknowledge the ethical concerns of attributing higher costs to the same health outcome based on national origin. Therefore, we also consider using a constant value of a statistical life across all countries. We estimate the cost per DALY in the same way as in our baseline case, but we use a constant cost per DALY by scaling down the U.S. value of a statistical life by the ratio of world per capita income 2020 USD10,936^4^ to U.S. per capita income. In this case, optimal spending increases 30% to USD40.8 billion, social surplus increases to USD6.08 trillion and the rate of return on investment rises to 31.7%.

**Fig. 2:**
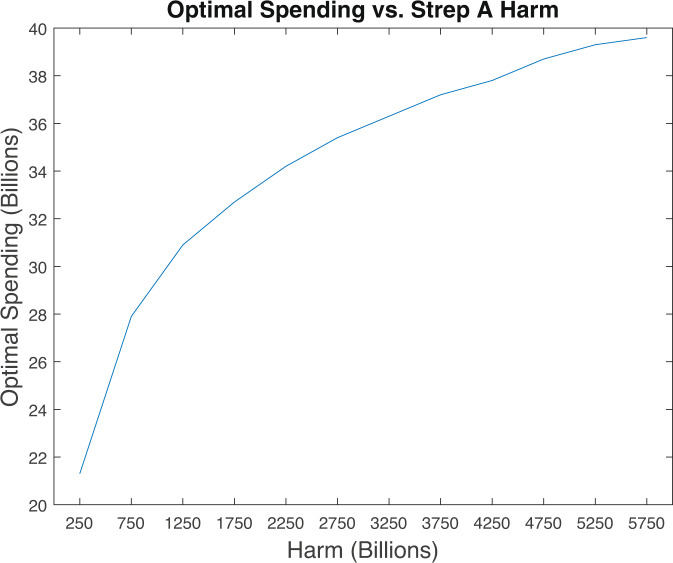
Optimal spending vs. Strep A harm. Monetary values are in 2020 USD.

Figure 2 explores broader sensitivity to the estimated amount of harm caused by Strep A globally. We graph optimal spending on a Strep A vaccine in USD billions vs. estimated harm caused by Strep A. Estimated harm ranges from USD250 billion to USD5.75 trillion. Clearly, optimal spending is rising in estimated harm. However, the relationship is not linear. Increasing estimated harm from our baseline value of USD1.85 trillion to USD5.75 trillion raises optimal spending only by about 20% to USD40 billion.

## Discussion

Optimal spending for Strep A research and development is large, in the tens of billions of USD. More importantly, the benefits are 50 times larger, ranging from USD1.6 trillion to USD37.9 trillion (in 2020 USD). Returns on investment range from 18% to 48.2% per year for 30 years. These returns are large compared with other interventions that have received considerable public support. For example, increased years of education are estimated to return about 9–10% per year in terms of increased income^5,6^.

Our results call upon national and international policy makers to fund and promote accelerated development of a Strep A vaccine. In this section we discuss a rationale for these policies and some mechanisms at the government’s disposable to provide this funding.

For many reasons, private sector investment in Strep A research and development is unlikely to reach our optimal amounts. First, some research and development, e.g., basic research, is hard to patent and therefore is unlikely to provide an adequate return on investment for private capital. Second, the high required rate of return of pharmaceutical companies due, in part, to their ability to sell products with patent-protected monopolies, will often discourage investment in all but the most promising projects. Third, the probability of success of an individual project is small, resulting in insufficiently high returns to justify the R&D risk. In contrast, a large portfolio of many projects greatly reduces the risk of not developing a viable product.

Public sector policy can move investment toward the optimal amount. A simple way to do this is to directly fund vaccine R&D projects. On a large scale, this mechanism of funding greatly reduces the risk of vaccine R&D by spreading risk across many possible projects. To raise funds for such an investment, governments have several approaches at their disposal: increasing taxes, crowding out other government spending, and debt finance.

Debt finance is particularly appealing as it allows the government to better align the costs and benefits of vaccine development. Any vaccine R&D project is likely to see benefits many years into the future. Debt allows a government to borrow money and pay back the principal in the future after R&D benefits materialize. Moreover, advanced economies can currently borrow at real interest rates substantially lower than our calculated returns on investment.

An alternative to direct funding would be for the government to encourage a large investment fund that would invest in a bond to raise capital for private sector investment into vaccine R&D. Many private investors pooling resources would fund many vaccine R&D projects at the same time^7^. Profits from successful projects would then provide a return to the bond holders. The government could encourage the development of such a private fund with a guarantee on the principal investment. Such an approach would reduce the risk of vaccine development while providing a role for both the public and private sector in vaccine R&D.

Speeding up the regulatory process, while monitoring safety, would also increase R&D. The annual required return on investment for the pharmaceutical industry is estimated to be at least 8% if not substantially higher^8^. At this required return, a 2-year regulatory delay would require expected profits be 16% higher to justify a pharmaceutical company making an investment. Reducing time to market would increase the number of projects the private sector finds viable, raising R&D expenditures of the private sector.

Finally, the full benefits of vaccine development cannot be achieved without equitable access to the vaccine. This point is especially notable with regard to Strep A, as most deaths occur in low-income countries due to lack of access to antibiotics. We expect that a Strep A vaccine will be made available in low-income countries in a way similar to pneumococcal and rotavirus vaccines. High-income countries should donate to international organizations like Gavi, the Vaccine Alliance; the World Health Organization; or UNICEF to support vaccine purchases for low-income countries. Such a policy would not be purely altruistic. Overuse of antibiotics is a key cause of increased antimicrobial resistance. Ensuring global access to an effective Strep A vaccine would be a potent defense against the development of such resistance.

Moreover, previous research has shown these donations to be a particularly effective form of foreign aid^9^. The GAVI model is effective because pharmaceutical manufacturers make substantial profits in high-income countries and then sell at discounts to low-income countries. Moreover, financing from the International Finance Facility for Immunization may be an additional way to support vaccine financing along with nontraditional debt finance that conditions on outcomes^10^. Potentially, a developing country manufacturer may also sell a Strep A vaccine, at a substantially lower price, as has happened with pneumococcal vaccines.

Our work has limitations. We calculate social surplus but do not analyze pricing. As a result, we cannot predict how the surplus will be split between increased population health and profits to manufacturers.

Additionally, while we view our model as an important step to understand the optimal level of investment, it abstracts from some aspects of the R&D process. We neglect the dynamic aspects of R&D, e.g., the possibility to learn from previous projects to either increase or decrease R&D. To fully understand how our results would differ in this dynamic context we would need to analyze a fully dynamic model. Such a model is beyond the scope of this paper; however, we can speculate, in part, how our results would differ in a dynamic environment.

One point is clear. In a dynamic context, the funder can observe past successes and then condition additional funding on these past successes. One possibility is that the funder will get lucky, with more successes than expected, and need to spend less. However, the funder may get unlucky, see many failures, and need to spend more than expected to get the same result. In this case, we view spending as a random variable, based on the random outcomes of the funded projects, with our model giving the expected spending that is needed on average.

A dynamic model also raises the possibility that the funder can learn about the probability of success from outcomes and then alter funding plans based on this new information. While giving more funding to approaches that have produced successes in the past may seem intuitive, the funder may also wish to reduce funding after successes because less harm from Strep A would be expected to remain. Learning should have a large effect in a model with many different approaches to develop a vaccine, all with highly uncertain probabilities. In this case, funding a few projects to learn about how likely the approaches are to succeed and then concentrating investment in the most promising approaches would be a useful strategy. We take comfort in the fact that Strep A vaccine development, according to our calibration, is a problem with few approaches, each with an estimated high success probability, and therefore we expect learning will not largely change the conclusions of our model.

Finally, as funding is spread out over multiple years in a dynamic model, this difference would delay the time required to develop a vaccine. The funder would need to wait for the various results to make additional funding decisions. These delays would also reduce rates of return as the time between making the investment and realizing the benefits would be extended. We have accommodated for this effect in our calculations by assuming a 10-year delay between investment and vaccine benefits and then assuming total benefits are spread out over a 30-year period. But the exact timeline of investment, discovery, and realization of benefits is uncertain.

An astute reader will notice that we omit the cost of manufacturing and delivery that would be necessary to realize the full health benefits. To understand the quantitative importance of this omission we obtain vaccine delivery costs from^11^. These costs are estimated to be USD3.70 per person vaccinated with a two-dose vaccine. Moreover, based on the UNICEF price for PCV-13 applicable to GAVI countries, USD3.30 per dose, we estimate an upper bound on manufacturing costs of USD6.60 per person vaccinated.^12^. We assume manufacturers do not sell below production cost and view this as a reasonable assumption given evidence that the manufacturing costs of Gardasil (an HPV vaccine) are possibly below the GAVI price^13^. Combining these costs, we estimate a manufacturing and delivery cost of USD10.30 per person vaccinated. We then calculate the present discounted value of these costs and compare them with the estimated health benefits. We find the present discounted value of these costs to be USD42 billion, which is 2.6% of our total estimated benefits. Consequently, we view our main conclusions as robust to the inclusion of manufacturing and delivery costs.

In calibrating the fraction by which the vaccine reduces harm we have not accounted for potential adverse effects of the vaccine. To explore the potential magnitude of these adverse effects we obtain data from the U.S. National Vaccine Injury Compensation Program^14^. The program has paid USD4.9 billion in total for 9304 claims. Therefore, it has paid out USD527,000 per claim. The program estimates it pays out about one claim per million vaccinations. Consequently, it has paid out USD0.53 per vaccination. We use this as a measure of the expected cost of adverse events per vaccine, and we calculate the present discounted value of these expected costs. This value is USD2.2 billion, which represents 0.14% of our total estimated benefits.

While additional vaccine costs and adverse events would lower our estimated benefits slightly, there are also important reasons to believe we have underestimated the benefits. For example, a Strep A vaccine may reduce the use of antibiotics, which is an important factor in the development of antimicrobial resistance. Additionally, it may prevent future health problems of an initial Strep A infection (e.g., future heart failure) that are not directly caused by Strep A and therefore not included in our benefits. Finally, the broad benefits of vaccines, e.g., increased labor force participation and productivity and increased education are omitted from our estimates^15^.

Potentially, adverse effects may be a larger concern with a Strep A vaccine than with other vaccines. This possibility is due to the U.S. Food and Drug Administration’s ban on Strep A vaccine human trials. However, this ban was based on one vaccine trial, from 1969, and the concerns raised by that trial have subsequently been questioned. In fact, in lifting the ban the U.S. Food and Drug Administration referred to its previous viewpoint as “obsolete,” and recent vaccine research has resumed with no similar adverse events^16^. We expect modern trials to perform extensive safety testing, and therefore in the calibration of our model we use a high cost to develop an approved vaccine of USD1 billion.

Our model takes harm from Strep A as given, and a vaccine as the correct mechanism to alleviate this harm. Scale-up of antibiotic use may provide an alternative approach to reduce the burden of Strep A. However, in our view, antibiotic scale-up has important limitations. Typically, antibiotic access is limited in low-income countries. This limited access stems from the inability to afford a visit to a doctor to obtain a diagnosis, the inability to afford a complete course of the antibiotic, and the lack of rapid tests to confirm Strep A infection^17^. Moreover, these costs are incurred for every Strep A infection. In contrast, a vaccine requires fewer contacts with the health care system (one to three depending on the number of required doses), and patients are not usually required to pay for vaccines in low-income countries^18^. Moreover, we base our estimates of Strep A harm on the Australian experience, in part, because it is a country where antibiotics are widely available but Strep A still has a substantial burden. We view it, therefore, as a rough estimate of Strep A harm even after a substantial scale-up of antibiotics. Finally, any global scale-up of antibiotic use comes with the potential cost of illegitimate use of antibiotics leading to increased potential for antimicrobial resistance.

The COVID-19 pandemic made clear that a large public investment in a vaccine can unlock enormous benefits. This recognition raises the question: for what other pathogens can the feat be repeated? Strep A is a most promising answer.

## Methods

Our model extends and generalizes a framework previously applied to COVID-19 vaccines^19^. While the application in this paper is to Strep A vaccine R&D, we stress that the model is applicable to many diseases and various R&D projects. For example, in other work, we have applied this model to calculate optimal R&D spending to alleviate harm from Alzheimer’s disease and related dementias^20^.

### Model framework

The model includes *N* different approaches to developing a preventative intervention or treatment for a known disease. These approaches are indexed by *n* = {1, 2, …, *N*}. Each approach could develop at least one successful treatment or preventative intervention with probability *p*_*n*_. The probability that one approach can succeed in developing a successful treatment is independent of the probability that any of the other approaches will develop a successful treatment or preventative intervention.

Within each approach (*n*) are projects indexed by *j* = {1, 2, …, *J*}. The success probability for a given project is *p*_*n,j*_ = *p*_*n*_*p*_*j|n*_ where *p*_*j|n*_ is the probability that project *j*, under approach *n*, succeeds conditional on approach *n* succeeding. This conditional probability of success is independent of the probability of success of any other project.

Each approach can reduce the harm caused by the disease by a fraction *Δ*_*n*_. This fraction represents the maximum harm reduction for the approach no matter how many of the approach’s projects succeed. The *Δ*_*n*_ sum to less than or equal to one. Moreover, harm is partitioned so that approaches 2 through *N* cannot address the first fraction of harm *Δ*_*1*_, approaches 1,…,3,4,5,…,*N* cannot address the fraction of harm *Δ*_*2*_ and so on. This simplifying assumption makes finding a numerical solution to the model substantially easier.

**Fig. 3:**
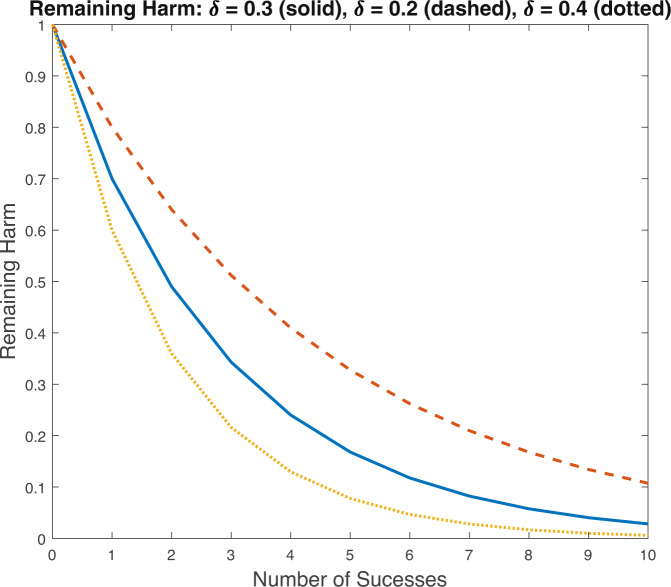
Remaining Harm. This figure plots remaining harm versus the number of successes for varying assumption about (δ) the fraction of remaining harm each success aleviates.

Each successful project under approach *n* alleviates the remaining expected harm by a fraction *δ*_*n*_. Expected remaining harm is calculated as the expected amount of harm that will remain from the disease, given the current level of funding for the given approach. Remaining expected harm is less than total harm because once projects have been funded a probability exists that prior projects will succeed and alleviate some harm from the disease. Figure 3 graphs the fraction of remaining harm as a function of the number of successes for differing values of *δ*_*n*_. The figure shows that with a *δ*_*n*_ = 40% almost no harm remains after 10 successes while with a *δ*_*n*_ = 20%, more than 10% of harm will remain after 10 successes.

Finally, funding a project requires a constant cost *c*.

We consider the perspective of a supranational organization that must choose which projects to fund from the universe of available projects. If *Ω* is a list of funded projects, then the organization’s problem is to choose *Ω* to maximize

1 $$E\left\{ {{\mathrm{Benefits}}\left( \Omega \right) - {\mathrm{Costs}}\left( \Omega \right)} \right\} = H\mathop {\sum}\limits_n {{{\Delta }}_nE\left( {1 - \Psi _n} \right) - c \times {\mathrm{num}}\left( \Omega \right).}$$

Here *H* is total harm caused by the disease, $\left( {1 - \Psi _n} \right)$ is harm depreciated by approach *n* and is equal to (1 − *δ*_*n*_)^*s*^ where *s* is the number of projects funded under approach *n* that are successful and num(*Ω*) is the total number of projects in the list *Ω*. *E* is the expected value operator.

To solve the model, we find the highest marginal (incremental) benefit project and add it to the list of funded projects as long as the marginal benefit is greater than the cost *c*. If more than one project has a marginal benefit equal to the highest marginal benefit, we choose the project with the lowest project number and then the lowest approach number.

Given a list (*Ω*’) of already funded projects, then, conditional on this list, the marginal benefit of funding project (*j*) using approach (*n*) that has not yet been funded is

2 $$mb_{j,n} = H \ast \Delta _n \ast E\left( {1 - \Psi _n} \right) \ast p_n \ast p_{j|n} \ast \delta _{n.}$$

In words, this equation means that the marginal (or incremental) benefit of funding the project is the expected remaining harm from the disease that is treatable with approach *n*, times the probability the project succeeds, times the fraction of harm reduced from the project’s success. Expected remaining harm from the disease that is treatable with approach *n* equals $H\Delta _n$ times the expected amount of harm alleviated from past successes. The expected amount of harm alleviated from past successes is given by $E\left( {1 - \Psi _n} \right) = \mathop {\sum}\nolimits_{s = 0}^S {\left( {1 - \delta _n} \right)^sP\left( s \right)}$, where *S* is the number of projects of approach *n* in *Ω*’, and *P*(*s*), the probability of obtaining *s* successes, is given by the binomial distribution with success probability $p_{j|n}$ and number of Bernoulli trials T equal to the number of projects *S*.

The total set of projects to be funded is chosen by sequentially adding the highest marginal benefit project to the list of funded projects until the maximum marginal benefit over unfunded projects, *mb*_*j,n*_, is less than the cost of funding the project *c*.

Finally, we can calculate total spending = total projects funded × *c*.

Our model is a static model. This means that the organization pays a one-time R&D cost and realizes a one-time benefit. However, to better match the dynamic nature of R&D investment we add two dynamic aspects to our calculations of total Strep A harm and R&D returns. First, to estimate the total harm from the disease we calculate harm for 100 future birth cohorts and calculate the present discounted value of this harm as our measure of total harm. Second, to estimate rates of return we assume that benefits from R&D investment begin 10 years after R&D costs are paid and that these benefits accrue evenly over a 30-year period.

### Data inputs and calibration

The model takes as inputs various parameters that need to be calibrated. Table 2 contains all values and the sources for our parameters. We assume two approaches are available to develop a Strep A vaccine: the M-protein approach and a catch-all other approach. We also consider sensitivity to four approaches, dividing the M-protein approach into three sub-approaches (N-terminal, minimal epitope, C-repeat epitope)^21^. Based on consultation with industry experts we calibrate the probability that an approach could succeed at 90% and use this same probability for all approaches. Industry experts argued that to receive funding for R&D under these approaches a high likelihood they could eventually succeed would be needed. (Our industry experts include Steven Black, Emeritus Professor of Pediatrics at the University of Cincinnati and Children’s Hospital; David Kaslow, Chief Scientific Officer at PATH; Bill Hausdorff, Lead, Vaccines Public Health Value Proposition at PATH; Andrew Steer, Professor and Pediatric Infectious Diseases Physician at the Murdoch Children’s Research Institute and the Royal Children’s Hospital Melbourne; and Jim Wassil, Chief Operating Officer at Vaxcyte. While we have consulted with these experts, responsibility for the content of this article is the authors’ alone).

**Table 2:** Parameter calibration.

Parameter	Value	Source
Number of approaches	2	^21^
Approach success probability	90%	Expert consultation
Project success probability	15%	^22,23^
Fraction harm reduction	30%	^24^
Strep A harm	USD1.85 trillion	^24^, authors’ calculations
Project cost inclusive of failures	USD1 billion	^26–28^

This table lists the parameters used in the calibration of the vaccine R&D model and the sources for these parameters. Monetary values in 2020 USD.

We estimate that a single research project has a 15% chance of resulting in an approved vaccine. Previous research has estimated very high success probabilities for prior vaccine projects: 33.4% and 22%, respectively^22,23^. We use a lower estimate here due to (i) selection bias—easier vaccine development projects are more likely to be attempted and therefore observed and (ii) the perspective of our consulted industry experts.

Based on the estimated value of a Strep A vaccine for Australia, we calibrate the fraction of Strep A harm that a single approved vaccine can eliminate at 30%^24^. While one would expect the efficacy of an approved Strep A vaccine to be substantially higher than 30%, this lower value reflects the fact that the vaccine is unlikely to be approved for all populations. Consequently, the amount of Strep A harm a vaccine can reduce is more accurately measured as efficacy multiplied by the fraction of the population for which the vaccine will be approved. Furthermore, this parameter also reflects the fact that vaccine coverage is incomplete and that harm reduction should be estimated net of any adverse events.

Using estimates for Australia, we find total global harm caused by Strep A to be USD1.85 trillion (in 2020 USD). This estimate is obtained by extrapolating estimated harm for the non-Indigenous Australian population to high- and upper-middle-income countries and estimated harm for the Indigenous Australian population to lower-middle- and low-income countries^24^. Table 3 describes this calculation in detail. A monetary value for Strep A harm is obtained by estimating disability-adjusted life years (DALYs) caused by Strep A and using value of a statistical life year estimates (VSLYs) to convert the DALYs to monetary values. Additionally, we include estimates of the economic burden, defined as the direct cost to the health care system, from Strep A–induced illness as an additional contributor to the dollar value of harm.

**Table 3:** Global Strep A harm calculation.

Australia		Birth cohort	DALYs	Economic burden 2015 $AU	DALYs per capita	Economic burden per capita
	Indigenous	18,537	739	18,247,362	0.04	984.38
	Non-Indigenous	286,840	4877	30,472,046	0.017	106.23
World (2020)		Birth cohort (thousands)	DALYs	Cost per DALY USD	DALY cost (2020 USD billions)	Economic burden (2020 USD billions)
	High income	45,639	775,978	53,926	41.8	3.98
	Low income	89,678	3,575,112	4457	15.9	6.00
Strep A harm		Discount rate	Cohorts		PDV harm (USD billions)	
	High income	3%	100		1119.8	
	Low income	3%	100		732.3	
				Total	1852.1	

This table calculates global harm from Strep A as the total expected, discounted harm for all birth cohorts born in 2020–2119 in the absence of a Strep A vaccine. We use estimates from Table 1 of ref. ^24^ to calculate per capita economic burdens (direct costs to the health system) and disability-adjusted life years (DALYs) caused by Strep A for the Indigenous and non-Indigenous population of Australia. We then use population estimates for ages 0–4 from ref. ^29^ by World Bank income category. We group high income and upper-middle income into high income and lower-middle income and low income into low income. We obtain the birth cohort size by dividing by 5. We calculate a global number of DALYs by multiplying these population numbers by the per capita estimates from Australia. The table shows this calculation for 2022 and we use the World Population Prospect projections for future cohorts. We estimate a cost per DALY for high-income countries by taking a U.S. value of a statistical life of 10 million^30^ and multiplying by the ratio of the World Bank per capita high-income cutoff (12,535 2020 USD) to per capita income in the United States (56,833 2020 USD)^31^ and dividing by life expectancy of 40.9 years at age 40^32^. We scale down this cost per DALY by the ratio of the World Bank low-income cutoff value (1036 2020 USD) to high-income cutoff value (12,535) for low-income countries. We calculate the economic burden for both high-income and low-income countries by multiplying the per capita economic burden by the birth cohort size. We convert from $AU using the 2015 USD exchange rate, 0.7522^33^, and to 2020 USD using the percentage change in the Consumer Price Index from 2015 to 2020, 9.2%^34^. For low-income countries we also multiply by the ratio of the World Bank low-income cutoff to high-income cutoff to adjust for lower health care costs in low-income countries. Finally, we calculate total harm by summing across 100 future birth cohorts and discounting with a discount rate of 3%. PDV stands for present discounted value. As the World Populations Prospects Data ends in 2100, we use average population growth rates to estimate population for the final 19 cohorts.

We rely on these Australian estimates for various reasons. First, the study is a high-quality study that examines the wide spectrum of Strep A disease manifestations. Second, the study gives an economic cost of Strep A disease, which is a necessary input into our model. Third, the Indigenous population has higher disease incidence and less access to health care, consistent with the experience of many in lower-income countries. In fact, as this study points out, the Australian Indigenous population experiences rates of Strep A-induced disease that are similar to lower-middle-income countries. This result is consistent with other studies that have shown the rate of rheumatic heart disease for Indigenous Australian and New Zealanders is between that of Sub-Saharan Africa and South-central Asia^25^. Fourth, because Australia has ready access to antibiotics, our extrapolation gives us a plausible approximation as to what total harm from Strep A would be even after a global scale-up of access to antibiotics.

However, we admit that we may underestimate global harm if the Indigenous population in Australia has better access to healthcare than the population in lower-income countries. To address this concern, we use alternative estimates of the global burden of Strep A disease, described in the results section. In this sensitivity check, we find that optimal spending and rates of return are notably larger than our baseline estimates. We therefore view our extrapolation as a conservative way to estimate the Strep A burden that a vaccine would address.

Finally, we take the cost of developing a successful vaccine, inclusive of failures, to be USD1 billion (in 2020 USD)^26,27^. This number reflects the development costs of previous vaccines, including the development of RotaTeq, a vaccine against rotavirus, and the USD472 million spent by the U.S. government to develop the Moderna COVID-19 vaccine^28^. We have used a higher number for costs here than these studies indicate, as our industry experts expect that a Strep A phase 3 trial would be particularly costly due to the need to monitor for complications from inflammation of the heart. These costs would include both monitoring for the condition and increased trial size to be able to allow detection of rare side effects.

### Reporting summary

Further information on research design is available in the Nature Research Reporting Summary linked to this article.

## Supplementary information

REPORTING SUMMARY

## Data Availability

All data used in this article are publicly available at the websites included in the reference section and upon request to the corresponding author.

## Code availability

Computer codes and spreadsheets used to calculate results are available upon request to the corresponding author.
